# Memoir on Partial Tetanus

**Published:** 1822-08

**Authors:** Baron Larrey


ART. IV.—*Memoir on Partial Tetanus.* By Baron LARREY.

THE neuralgiæ consist in inflammation, either in the nerves of the encephalon or of the spinal marrow. They are characterised by tearing pains, which are propagated to the most remote ramifications of the nerves affected, and are commonly accompanied by convulsive movements in the muscles to which they belong. The sensibility and temperature of the parts are also increased, with redness and swelling, during the paroxysm, and the symptoms are liable to intermissions, which vary according to circumstances.

Such is the ordinary course of these affections, but they present infinite anomalies. I am not aware that the singular phenomena which were witnessed in the subjects of the following narrative, have been observed by other practitioners.

The subject of the first was a young soldier, who received a violent kick in the forehead from a horse. He was knocked down by the blow, and received a wound in the right eye-lid, occupying the whole line of the upper margin of the orbit. By attentive examination, a fracture was discovered in the external lamina of the frontal sinus, with splinters depressed in it. The eye of this side projected under the eye-lids, which were closed and swoln by a deep ecchymosis extending over the integuments of the face and left eye-lid. The man was insensible, bled copiously from the nose at the time of the accident, and continued to void blood by the right nostril. Some hours afterward, when his sensibility had returned, he complained of acute pains in the wound, and had convulsive movements in the lips and lower jaw.

On my first visit the morning after the accident, I found the man in the state which I have described. I freely dilated the contused and lacerated wound, and extracted from the frontal sinus a considerable quantity of black and coagulated blood, with several splinters imbedded in it. The sinus was then laid open. Air, during inspiration, came from the wound, and blood flowed from the nostrils. The dressing was made by means of soft lint and rag spread with styrac ointment, over which were applied compresses and retaining bandages. A few hours afterwards, he was bled in the temporal artery; was cupped in the nape of the neck and between the shoulders; and mustard baths of the legs, with a diluting antiphlogistic regimen, were prescribed. During the first day, all was calm; but the mental faculties were disturbed, and the memory had entirely disappeared. The bleeding was repeated; the diluents and mustard baths were persisted in; nevertheless, strong symptoms of frenzy supervened, with fever, delirium, convulsions, and coma, which terminated the existence of the patient on the nineteenth day of the accident.

Although the dressings were effected with great care, they caused intense pain in the head, and generally produced convulsive movements in all the muscles of the face, the neck, and the arm of the right side; while the left was insensible, and half paralyzed.

Twenty-four hours after death, the body was opened. We found considerable inflammation, with swelling of the membranes of the frontal sinus and nasal fossæ. A fissure was also perceived at the posterior part of the sinus, with marked inflammation of the corresponding portion of the dura mater. An effusion of blood and serum had taken place, between this membrane and the right anterior lobe of the brain. The pia mater was equally inflamed to a great extent, and studded with points of suppuration. The substance of the encephalon, and especially that of the right hemisphere, were dense, and their vessels were turgid. A large quantity of reddish serosity was accumulated in the ventricles. The mucous membranes of the larynx were red and inflamed; the bronchiæ were filled with reddish mucosities; and the lungs were gorged, and of a brown colour. The abdominal viscera were healthy.

The rapid march of these inflammatory symptoms at first induced me to believe that the extreme sensibility of the pituitary membrane had greatly contributed to their development; but the following cases proved to me that the cause of the symptoms was rather to be ascribed to the fissure of the frontal bone, and to the laceration of the fibrous tissue of the meninges of the brain. Nevertheless, I believe that the state of the pituitary membrane contributed, sympathetically, to aggravate that of the meninges; since the same inflammation was found in the whole of the air passages. It was not without reason that the ancients considered wounds of the frontal sinus to be attended with great danger, especially when the air passed into the nasal fossæ; on which account they recommended the careful occlusion of these wounds.

The subject of the second case was a cuirassier of the second regiment of the Guard, a man of dark countenance, an athletic constitution, who, on the 21st of January, 1821, received, on the whole line of the superior orbitary margin of the right eye, a kick from his horse. The blow was so violent, that the external table of the frontal sinus was broken in pieces, and the man was thrown backwards in a state of insensibility, remaining rigid as a corpse. He was found covered with blood, which had flowed from his nose and ears. The regimental surgeon, after having made a provisional dressing, transported the patient to the hospital, where he arrived in the middle of the night. At my visit in the morning, he was quite insensible. The head was strongly turned to the right side. The eye, on this side, was forced out of the orbit, forming with the eye-lids a prodigious projection, and blackened by a strong ecchymosis, which extended over the face and forehead. The right side of the body was affected with tetanic rigidity; the skin was cold, and appearances announced approaching death. I caused the head to be shaved, and warm embrocations of camphorated vinegar to be applied to the whole body. I dilated the wound freely in all directions, thereby exposing the external plate of the right frontal sinus, which was reduced to splinters; the most moveable of which, with some clots of blood, I extracted. During the incisions, so much blood was voided that it became necessary to tie the branches of the temporal artery, which had been divided. The first dressing was hardly completed before the man, having recovered his faculties, related to us the manner of the accident; and from this time he has mixed in the conversation of his comrades. I prescribed for him diluting drinks, with stimulating glysters, mustard baths, and the application of ice to the head. He passed the remainder of the day with tranquillity; but the skin having become hot, and the pulse accelerated, the surgeon of the guard judged it proper to bleed him, according to instructions which I had left.* Notwithstanding this treatment, the night was greatly disturbed. The patient complained incessantly of intense pains, which had begun in the occiput and region of the wound, immediately after his recovery from the lethargy into which he had been plunged by the accident; but the medical officer belonging to the guard, fearing a renewal of hemorrhage, thought it imprudent to remove the dressing, and confined himself to the application of ice and the use of the antispasmodic sedatives I had prescribed. On the 23d, the occipital pains having increased, and the irritation being considerable, I caused him to be cupped on the right side of the neck, between the shoulders. The temporal artery was opened, and the ecchymosed parts were scarified. Rag, covered with ointment of styrax, with some soft lint, were placed upon the wound of the sinus, and an aromatic cataplasm over the temporal artery. The application of ice to the head and the use of diluting and anodyne drinks were persevered in, and bleeding in the arm was renewed; but the night was passed in great agitation. On the 24th, the pains in the back of the head were very acute; and, an œdematous point being perceived, I suspected fracture in the corresponding part of the occiput. A deep incision was made, by which I was enabled to feel the surface of the bone, but no depression or inequality was found.

* It is a great, and often a fatal, error to bleed immediately after an accident of this kind. The loss of blood increases the collapse, and often takes away the little resource left to the constitution for the re-establishment of the equilibrium in the vital functions.

I covered the incised part with cupping glasses: it yielded a considerable quantity of blood, and the patient was relieved. The usual dressings and remedies were continued.

The suppuration of the wound in the forehead became abundant, and voided itself through the nose. The ecchymosis gradually disappeared, but the patient was deprived of sight on the affected side. The pains in the occiput continued, and the patient began to experience numbness in the two limbs of the wounded side. Between the fifteenth and twentieth day, many small splinters, which had escaped our view, came away, and the wound healed; upon which, the pain in the occiput and temporal region immediately increased to a violent degree. Exquisite nervous sensibility supervened, and the limbs of the affected side were in a state of convulsion, which soon assumed the character of tetanus. The pains in the head became so intense, that he could not bear the slightest touch on these parts without piercing cries, accompanied by shudderings and convulsive movements. After these new symptoms, bleeding was renewed at intervals, in the jugular vein, the arm, and foot. Blood was taken from the nape of the neck, shoulders, and spine, by cupping. Ice was again applied to the head, and mustard baths to the feet. A momentary calm was produced by these means; but the symptoms incessantly recurred, and threatened the existence of the sufferer.

In this alternation of amendment and deterioration, he continued to the forty-first day, at which period the tetanic symptoms of the two limbs suddenly increased. The muscles of the shoulders, arm, and fore-arm, were forcibly retracted, prodigiously swoln, and renitent: those of the thigh and leg were similarly affected. The right testicle was swoln, and acutely painful. To our great surprise, the hairs of the mustachios of the right side stood on end, and could not bear the slightest touch, or the incision of the smallest number of hairs, without producing intense pain. This experiment was many times repeated, and uniformly with the same effect.

This extreme increase of the sensibility of all the tissues on the right side induced me to believe that a deep fissure existed either in the right occipital region or in the forehead, which had torn the dura mater, and had produced effusion immediately under this membrane, beneath the tentorium, or under the right lobe of the portion of the brain, as far as the entrance of the spinal canal;—that the consequence of this was inflammation, extending to the origin of the corresponding nerves of the medulla oblongata, and to that of the nerves of the same side of the spinal marrow, being propagated even to the substance of their principal branches. To this circumstance are we to ascribe the pain, numbness, tetanic contraction, swelling of the muscles, and increase of the sensibility in all the soft parts of the two corresponding members; while those of the opposite side, including the organ of intellect, remained uninjured. The mechanism of speech was impaired, and the sight of the right eye was nearly lost: the patient, in fact, scarcely perceived the light, which seemed speckled with sparks of fire. The iris had preserved its functions. The smell of the right side was gone, but the hearing was as perfect as on the other.

These phenomena appear to me to depend, first, for the tetanic affection, on inflammation and compression upon the cerebellum, and the portion of the medulla oblongata on the same side; because in this part of the encephalon, as Dr. GALL has observed, and as I have repeated in my Memoirs on Wounds of the Head, the nervous filaments of the brain do not intersect each other.* I find, in the same volume, p. 263, a case of wound in the head, similar to the above. A portion of the occipital bone was separated by a sabre at the battle of Benevento, and the right lobe of the brain was carried away. This wound was followed by the loss of sight and hearing of the right side; the right testicle was withered; and the extremities of the same side were paralyzed. Nevertheless, the patient gradually recovered; when, in consequence of intemperance, new inflammatory symptoms supervened, and increased with such rapidity, that he died tetanic on the twenty-ninth day. The pains of the wounded side of the head and dorsal spine were so intense, that he uttered the most piercing cries. He laid constantly on the right side, was motionless, and could not bear the slightest touch on the affected parts, or even suffer the cutting of a small portion of hair, without alarming syncope. On examination after death, the cerebellum was diminished in size, was firmer than natural, with signs of inflammation in the medulla oblongata and spinal marrow, as well as at the origin of the nerves of the encephalon, which were found withered.

A singular phenomenon presented itself in this man at the early period of his wound: a silver probe, passed down to the exposed brain, caused no pain, but instantly produced vertigo, syncope, and convulsion.

As to the anomalies of the cerebral functions, I should remark that the integrity of his mind was preserved, excepting as regards the memory of substantive nouns, which was for some time suspended. The disease was confined to the cerebellum of the right side, and to some points of the external border of the basis of the right hemisphere of the brain. The upper periphery of this lobe, where the intellectual organs are supposed to reside, remained untouched. The hearing, the sight, the

* *Campagnes*, vol. iv, p. 180.

left eye, the smell, and the taste of the same side, are in their natural state. The loss of sight and smell of the wounded side depends upon immediate injury of the nerves, or nervous membranes, of the corresponding organic portion. The impediment to speech, I think, arose from compression of the lingual nerve on the same side, at its origin or passage in the foramina of the basis of the cranium.

It is more difficult to account for the increased sensibility of the right side of the body, especially the pain from the cutting of the hair and mustachios, which could not be cut or touched in the slightest degree without causing shuddering, convulsions, and perspiration. Are the hairs themselves sensible? —this is hardly possible:—or is it the vibration occasioned by the touch, which is transmitted to their bulb or root, where the nervous filaments reside?—this is the most probable supposition. But I must leave to the physiologist the solution of this problem. The hairs neither changed their form nor size, but were somewhat darker than the hair of the left side of the head.

I entertained a hope that these symptoms would be transitory, and that they would readily yield to local depletion, revellents, antispasmodics, and anodyne diluents, taken inwardly. I first ordered general and local bleeding; a blister was applied to the right temple, caustics to the neck and mastoid region; while moxas and the actual cautery were used, in succession, to the neck and anterior part of the shoulder of the wounded side, with beneficial, but momentary, effect. The symptoms were incessantly renewed by the most trivial causes. The contraction did not yield, in the slightest degree, under the influence of any of the means employed. I tried baths almost cold, and the prussic acid, so much recommended by Professor Tommassini. The first immersion in water was followed by tremors and syncope; nor could the bath be supported at any temperature. The use of emulsions, made with equal parts of sweet and bitter almonds, to four ounces of which was added from eight to ten drops of the distilled water of the lauro cerasus, produced febrile symptoms, violent colics, and dysentery, with constant tenesmus, and a sensible increase of the cerebral and other inflammatory traumatic symptoms, which had continued from the twenty-first day of the accident. The pulse remained almost natural, and the digestive functions were performed with ease.

Such was the condition of the patient till the middle of March, when all the inflammatory symptoms re-appeared, with a sensation as if the bones of the cranium were being drawn asunder by pincers; and the tetanic contraction of the limbs had increased to such a degree, that the extremities of the fingers were imbedded in the palms of the hands, without the possibility of preventing it. Venesection was repeated for the twenty-fourth or twenty-fifth time, and the local bleedings, perhaps, for the hundredth. Ice was again applied upon the head, and the use of mucilaginous and diluting drinks persisted in. The erectility of the hair, accompanied with the most exquisite sensibility, increased to such a degree, that the application of ice, or any cataplasm, could no longer be borne upon the head: we were, therefore, obliged to confine our applications to a retentive bandage, applied with moderate tightness, which produced slight relief. The wound of the forehead was healed on the forty-first day of the accident; but the ulcerations from the caustic were kept open in the nape and right side of the neck; and, in addition to the internal remedies before mentioned, embrocations of camphorated oil of chamomile were daily applied to the affected limbs.

The man remained in this situation until the 26th of April, 1821, when he was presented to the Medical Society of the Faculty of Paris.

The experiment of cutting the hair, although performed with excellent scissars, and without the knowledge of the patient, was followed by shuddering, convulsions, and painful tremblings, with distressing pricking in all the affected parts.

Notwithstanding this state of suffering, the man preserved his colour and embonpoint, because, in fact, the vital functions had experienced no alteration; unless we may except the momentary disturbance produced by the prussic acid, although administered in a very small dose.

At length the disease became stationary, and we were left in a state of uncertainty as to its termination, yet not without apprehensions of its fatality. The accession of hot weather produced, however, copious perspirations, which were followed by slight relaxation in the contracted parts, and the pains diminished in intensity. His speech became more free, and he could walk during great part of the day without experiencing bad symptoms. On the 18th of August, seven months after the accident, he was permitted to leave the hospital. He returned in the beginning of January. His hand, fore-arm, and under part of the arm, were now withered: although formerly so painful, they have become almost wholly insensible, and a marked diminution in the temperature is perceivable. The lower limb presents the same character, and the patient experiences cold to a degree of intensity that cannot be overcome by artificial heat.

When this species of atrophy arrives at its highest point, I have remarked that the functions of life are endangered. The patient wastes, and falls into a state of anxiety and melancholy so distressing, that he eagerly desires the amputation of the limb. It may become a question whether such an operation be really indicated, and whether the equilibrium of the other functions could be thereby re-established?*

*January 21, 1822.*

* *Journal Complementaire;* Mar. 1822.

